# Supplementary material for: TAXISCAN, Optimizing throughput and behavioral depth in standard C. elegans chemotaxis assays
Source: bioRxiv. 2026 Jul 25:2026.07.21.739905. Preprint. [Version 1] doi: 10.64898/2026.07.21.739905 (PMC13419531; doi:10.64898/2026.07.21.739905)
Supplement: 1 [file NIHPP2026.07.21.739905v1-supplement-1.pdf]

bioRxiv preprint doi: https://doi.org/10.64898/2026.07.21.739905; this version posted July 25, 2026. The copyright holder for this preprint (which was not certified by peer review) is the author/funder, who has granted bioRxiv a license to display the preprint in perpetuity. It is made available under a CC-BY 4.0 International license.

Supplemental

Supplemental Figure 1. Chemotaxis Plate Layout.

(A) Graphical representation depicting the design and layout of a 5 cm chemotaxis plate from a top-down view. **(B)** The same plate layout after processing using TAXISCAN, which captures plates from the bottom and crops the outer 0.5 cm border of the plate. Black X's are marked 1 cm from the edge of the plate in quadrants QI (red) and QIV to correspond to 2 μL of the test compound (Orange). Small black dots are marked in the same spots of quadrants QII and QIII to denote the 2 μL of buffer control (blue), and the dot at the center of the plate denotes where worms are added. **(C-D)** Example worm distributions and Chemotaxis Indexes (CI) in response to **(B)** an attractant or **(C)** a repellent added in the test quadrants. The dark grey borders represent the edge of the 5 cm plate, and the light grey borders represent the 4 cm viewable window TAXISCAN crops out of the original image. The dotted lines represent the relative quadrant boundaries and the 1 cm diameter neutral region.

Supplemental Figure 2. Scanner Setup.

(A) Epson V600 scanner with a single column of chemotaxis plates. **(B)** The original white removable cushion on the back of the scanner lid, which was replaced with **(C)** a black paper backing to improve contrast in the resultant scanned images.

Supplemental Figure 3. TAXISCAN Image Processing.

Representative images demonstrating TAXISCAN's processing of a column of chemotaxis plates. **(A)** A raw image captured using the Epson V600 scanner, taken from

bioRxiv preprint doi: https://doi.org/10.64898/2026.07.21.739905; this version posted July 25, 2026. The copyright holder for this preprint (which was not certified by peer review) is the author/funder, who has granted bioRxiv a license to display the preprint in perpetuity. It is made available under a CC-BY 4.0 International license.

the bottom of the plates at 1200 dpi. **(B)** TAXISCAN's plate recognition output, which defines the viewable region of the plates (red) and labels them (yellow text). **(C-E)** Downstream of a plate by **(C)** isolating and re-orienting, **(D)** segmenting the worms from the background, and **(E)** scoring by worm size and location. X's denote where 2 µL of the compound was added, and worms are labelled by whether one (red), two (blue), three (green), or a large group (yellow) were detected. The plates are further divided into quadrants (black axes) and a 1 cm diameter neutral region (black circle), with *n* worms detected in each region (white). The Chemotaxis Index (CI) for the plate ranges between -1 and +1, representing total avoidance and attractance, respectively.

Supplemental Figure 4. Accuracy in Worm Detection.

Correlation and residual plots comparing the total number of worms detected on a plate by different scoring methods, clustered by if < 100 (red) or > 100 (blue) worms were detected on the plate. **(A, D)** Counting worms a plate under a microscope by hand (Standard Scoring) versus a scanned image of the same plate (Manually Scoring Images), with separate correlation models for each cluster. **(B-C, E-F)** Counting worms via TAXISCAN versus **(B, E)** Standard Scoring or **(C, F)** Manually Scoring Images. The correlation model (dark blue line) fit is given by the R^2, p-value (P), and 95% confidence interval (grey shaded region), with slope=1 for reference (grey dashed line). Clustering by worm count was performed via k-means clustering.

Supplemental Figure 5. Accuracy in Chemotaxis Index Determination.

Correlation and residual plots comparing the Chemotaxis Index calculated for a plate by different scoring methods, clustered by if < 100 (red) or > 100 (blue) worms were detected

bioRxiv preprint doi: https://doi.org/10.64898/2026.07.21.739905; this version posted July 25, 2026. The copyright holder for this preprint (which was not certified by peer review) is the author/funder, who has granted bioRxiv a license to display the preprint in perpetuity. It is made available under a CC-BY 4.0 International license.

on the plate. **(A, D)** Counting worms on a plate under a microscope by hand (Standard Scoring) versus a scanned image of the same plate (Manually Scoring Images), with separate correlation models for each cluster. **(B-C, E-F)** Counting worms via TAXISCAN versus **(B, E)** Standard Scoring or **(C, F)** Manually Scoring Images. The correlation model (dark blue line) fit is given by the R^2, p-value (P), and 95% confidence interval (grey shaded region), with slope=1 for reference (grey dashed line). Clustering by worm count was performed via k-means clustering.

Supplemental Figure 6. Impacts of Chemical Stimuli and Population Size on Worm Grouping Patterns.

The percentage of wildtype (N2) worms that formed groups of 3 or more out of the total number of worms detected on a chemotaxis plate, sorted by **(A)** small and **(B)** large plate populations. Worms were exposed to 1% IAA (green) 10% 1-octanol in 50% ethanol in 50% ethanol (blue), 100 mM $CuSO_4$ (purple), or chemotaxis buffer as a negative control (Null, red) with their respective buffers in the control quadrants of the plates. Error bars are SEM. Plate population sizes are separated into <100 (red) or >100 (blue) worms per plate, determined via k-means clustering.

Supplemental Figure 7. Wildtype Migration Over Time.

The kernel density estimate (Worm Density, left y-axis) for individual worm travel distances (Distances, x-axis) over time (right y-axis). Worms were exposed to 1% isoamyl alcohol (IAA, green) in 50% ethanol, 10% 1-octanol (blue) in 50% ethanol, 100 mM $CuSO_4$ (purple), or chemotaxis buffer as a negative control (Null, red). Vertical lines indicate the

bioRxiv preprint doi: https://doi.org/10.64898/2026.07.21.739905; this version posted July 25, 2026. The copyright holder for this preprint (which was not certified by peer review) is the author/funder, who has granted bioRxiv a license to display the preprint in perpetuity. It is made available under a CC-BY 4.0 International license.

approximate means of the bimodal peaks detected via standard EM algorithm. The center of the plate is located at x = 0 mm, and the compound is located at x = 15 mm.

Supplemental Figure 8. *slo-1* Mutant Migration Over Time.

The kernel density estimate (Worm Density, y-axis) of the percentage of individual *slo-1* mutant worm travel distances from the center of the plate (Distances, x-axis) over time in response to chemotaxis buffer alone. Chemotaxis behavior metric readouts comparing wildtype (N2, red) worms to *slo-1(ky389)* gain-of-function (GoF, green), *slo-1(js379)* loss-of-function (LoF, blue), and *slo-1(gk602291)* partial loss-of-function (pLoF, purple) mutants. Vertical lines indicate the means of the bimodal peaks detected via standard EM algorithm. The center of the plate is located at x = 0 mm, and the compound is located at x = 15 mm.

Supplemental Figure 9. *slo-1* Mutant Chemotaxis.

Representative chemotaxis images for Wildtype (N2, red) worms and *slo-1* partial loss-of-function (*slo-1(gk602291)*, green), gain-of-function (*slo-1(ky389gf)*, blue), and loss-of-function (*slo-1(js379lf)*, purple) mutants. Worms were exposed to 1% isoamyl alcohol (IAA) in 50% ethanol, 10% 1-octanol (Octanol) in 50% ethanol, 100 mM $CuSO_4$, or chemotaxis buffer as a negative control (Null). X's denote where 2 μL of the compound was added, and worms are labelled by whether one (red), two (blue), three (green), or multiple (≥3 worms, yellow) worms were detected in a group.
